# Special Issue “Investigation of Polymer Nanocomposites’ Performance”

**DOI:** 10.3390/molecules27041180

**Published:** 2022-02-10

**Authors:** Andrea Dorigato, Giulia Fredi

**Affiliations:** Department of Industrial Engineering and INSTM Research Unit, University of Trento, Via Sommarive 9, 38123 Trento, Italy

## 1. Introduction

Through this editorial, we aim to present the main aspects related to the scientific contributions that can be found in the Special Issue dedicated to the topic “Investigation of Polymer Nanocomposites’ Performance”. By going through this editorial, the readers will have an idea of the potential of polymer nanocomposites in some cutting-edge applications.

It is well-known that nanotechnology represents one of the most promising areas of current research and development in several fields. In the case of polymer science, the investigations have been mainly focused on novel technologies such as nanoelectronics, polymer-based biomaterials, nanoparticle drug delivery, miniemulsion particles, layer-by-layer self-assembled polymer films, electrospun nanofibers, imprint lithography, polymer blends, and nanocomposites [1,2]. For polymer nanocomposites, great attention has been devoted in the past decade to issues regarding the enhancement of the mechanical properties, but, in our opinion, other aspects should also be highlighted, such as gas- and UV-barrier properties, fire resistance, electrical and magnetic properties, and polymer blend compatibilization. In fact, the main interest in polymer nanocomposites is the possibility of tailoring the properties of polymer matrices with minimum filler contents, thereby obtaining multifunctional materials endowed with physical properties not achievable with traditional micrometric fillers [3,4]. To have a better comprehension of the aspects governing the physical response of these novel materials, the elaboration of innovative experimental approaches is of utmost importance. Therefore, the main aim of this Special Issue is to create an open forum where researchers may present innovative approaches to characterize and model the multifunctional properties of polymer nanocomposites. For this reason, the contributions to this Special Issue, in the form of both original research and review articles, cover the characterization and modeling of the most important physical properties of these materials, e.g., microstructural features; mechanical and viscoelastic behavior; thermal, electrical, and barrier properties; and antibacterial behaviour.

After the editorial process of analysis and in-depth verification by reviewers, six contributions were accepted, coming from researchers working in universities located in five different countries.

## 2. The Contribution of This Special Issue

The articles included in this Special Issue focus on the importance of polymer nanocomposites in some emerging technical fields. This Special Issue reports only some examples of the potential of nanocomposites in modern technology. Given the complexity of the discussed topic and the wide variety of technological applications of these materials, we have not been very restrictive in setting specific themes because nanocomposite technology can be approached from a multitude of angles.

Given the recent environmental concerns about plastic pollution, in the last years, there has been a worldwide increase in societal interest in using environmentally friendly plastic materials, which has in turn increased the number of polymers being researched and developed. Among them, great attention has been devoted to bioplastics, i.e., plastics derived from renewable resources and/or biodegradable materials [5]. These materials represent a promising alternative to traditional petroleum-derived polymers. Not only can bioplastics show alternative disposal pathways, thus limiting the amount of plastic waste ending up in our environment, but they also allow a considerable reduction in the carbon footprint throughout the whole life cycle. These inherent advantages have raised academic and industrial interest in bioplastics, and substantial efforts have been made to translate their intrinsic benefits into sustainable applications that are equally or more efficient than those involving traditional plastics. This is the reason why, in this Special Issue, many papers have been focused on bioplastic-based nanocomposites. 

The paper of Calambas et al., entitled “Physical-Mechanical Behavior and Water-Barrier Properties of Biopolymers-Clay Nanocomposites”, reports on the preparation of novel biodegradable starch-polyvinylalcohol (PVA)-nanoclay nanocomposites films [6]. Different nanofiller amounts were dispersed within the polymer matrix by solvent casting, and the dispersion was performed by magnetic stirring and sonication. It was demonstrated that the addition of a small nanoclay amount (up to 0.5% *w*/*v*) can strongly improve the barrier properties and the tensile mechanical behavior of the resulting films. The materials could be therefore considered as good candidates for the production of novel biodegradable packaging films.

In the last decades, bioderived polysaccharides, such as chitosan (CS) and pectin (PC), have attracted the attention of researchers and industries, as they can be applied in the biomedical and packaging field [7]. Moreover, they can be promising candidates for the development of sustainable films and coatings for cultural heritage protection, as highlighted in the paper of Infurna et al., entitled “Understanding the Effects of Crosslinking and Reinforcement Agents on the Performance and Durability of Biopolymer Films for Cultural Heritage Protection” [8]. In order to improve the physical behavior of these chitosan, pectin, and chitosan/pectin films, the authors proposed the addition of natural crosslinking and reinforcement agents, such as citric acid (CA) and halloysite nanotubes (HNTs). Both these agents showed beneficial effects on the wettability and the photo-oxidation resistance of these materials, which enables their applications as cultural heritage protection films.

Another interesting group of biopolymers that has recently attracted much academic interest is that of poly(alkylene furanoate)s (PAFs). These novel polymers can be synthesized from the polycondensation between an alkylene glycol and 2,5-furandicarboxylic acid (FDCA), which has been recently listed among the top 12 high-value-added chemicals from the biorefinery of carbohydrates by the United States Department of Energy. In this sense, PAFs represent the most promising bioderived alternative to fossil-based poly(alkylene terephthalates) (PATs), as they show thermo-mechanical and gas barrier properties similar or superior to those of the corresponding PATs [9]. The paper of Fredi et al., entitled “Multifunctionality of Reduced Graphene Oxide in Bioderived Polylactide/Poly(Dodecylene Furanoate) Nanocomposite Films”, reports the first attempt to prepare bioderived nanocomposite films by blending polylactic acid (PLA) and poly(dodecylene furanoate) (PDoF) and using reduced graphene oxide (rGO) as a multifunctional nanofiller [10]. The resulting PLA/PDoF blends were immiscible, but the addition of rGO promoted the dispersion of the secondary phase and the interfacial interaction, suggesting thus the role of this nanofiller as a blend compatibilizer. rGO also increased PLA crystallinity and the tensile strength, without significantly impairing the strain at break. Moreover, rGO decreased the electrical resistivity and the gas permeability of the films. This work stressed the positive and sometimes synergistic role of PDoF and rGO in tuning the physical properties of PLA, thus highlighting the promising properties of these novel bio-based nanocomposites for packaging applications.

Among the biocomposites containing small amounts of inorganic nanofillers, those including selenium nanoparticles (SeNPs) could be particularly interesting because of their high bioavailability, low toxicity, and therapeutic properties [11]. In fact, Selenium is a micronutrient with elevated biological activity, which could be extensively applied in the prevention and treatment of some diseases. In this sense, the introduction of SeNPs in biopolymers such as polysaccharides (e.g., fungal beta-glucans) could result in bioactive, biocompatible, and biodegradable composites with applications in the biomedical field. The potential of these systems is well described in the review of Tsivileva et al., entitled “Polymer Nanocomposites of Selenium Biofabricated Using Fungi”, which presents a comprehensive summary about the mycosynthesized selenium polymeric nanocomposites, the mechanisms of the involved processes at the chemical reaction level, and the future challenges in this field [12].

As discussed earlier, the scientific interest in polymer nanocomposites is not only aimed at improving the mechanical performance, but also at increasing the multifunctionality of the resulting materials. A clear example of this approach has been reported in the paper of Bertani et al., entitled “Improving the Antimicrobial and Mechanical Properties of Epoxy Resins via Nanomodification: An Overview”, which summarizes the potential of multifunctional nanomodified epoxies coupling improved antimicrobial activity and enhanced mechanical properties [13]. These materials could find wide applications such as antifouling and anti-corrosion coatings for marine environments, dental applications, and antimicrobial fabrics. This work discusses the different approaches to obtain antimicrobial activity, together with the methods used to evaluate their efficacy against bacteria and fungi. This paper also reports an analysis of the mechanical behavior of nanomodified epoxy with antimicrobial properties, regarding both nanofilled matrices and glass/epoxy composite laminates. The fracture toughness and the interlaminar properties of these multifunctional materials were comprehensively investigated.

The application of novel approaches for the formulation of multifunctional rubber-based nanocomposites could be particularly interesting for the modern tire industry. Considering that rubber materials generally exhibit poor thermal transfer, the addition of carbon-based or inorganic thermally conductive fillers at elevated amounts could represent an effective solution to achieve satisfactory heat dissipation performance [14]. However, this dramatically alters the mechanical behavior of the resulting materials, and a proper filler functionalization is thus often required. These issues are debated in the review proposed by Mirizzi et al., entitled “Tailoring the Thermal Conductivity of Rubber Nanocomposites by Inorganic Systems: Opportunities and Challenges for Their Application in Tires Formulation”, which summarizes the recent approaches applied to improve the thermal conductivity of rubber nanocomposites thanks to the addition of inorganic and hybrid filler systems [15]. This review analyzes how the structural and morphological features of the fillers influence the heat transfer behavior of the resulting nanocomposites, with the aim of providing valuable methodological tools for the design of thermally conductive rubber nanocomposites for the tire industry.

## 3. Conclusions

In our opinion, the purpose of this Special Issue has been well achieved. Going through the six papers grouped in this Special Issue, the readers can have some valuable examples of the real potential of polymer nanocomposites in modern society. Interestingly, in these works, the investigation of the physical behavior of the presented systems has been often accompanied by the definition of novel experimental protocols.

As a future goal, we would like to extend the proposed approach by editing another Special Issue in which the multifunctional properties of polymer nanocomposites could be applied to energy saving/storage/management applications. In fact, many examples of polymer nanocomposites for electrical and thermal energy storage can be found in the open literature, as well as novel nanocomposite systems for thermal insulation. Considering the recent environmental concerns about global warming, a Special Issue on this topic could meet the interest of many industrial and academic readers.
